# The relevance of sputum galectin-7 levels to clinical and prognostic factors in patients with chronic obstructive pulmonary disease: a prospective cohort study from China

**DOI:** 10.1186/s12890-026-04171-9

**Published:** 2026-02-27

**Authors:** Wenqiang He, Xueping Li, Haining Huang, Zitong Tang, Junqing Zhu, Yuqiong Yang, Zizheng Chen, Chengyu Miao, Huajing Yang, Shanshan Zha, Zifei Zhou, Jiachun Liu, Zhenyu Liang, Rongchang Chen, Fengyan Wang

**Affiliations:** 1 State Key Laboratory of Respiratory Disease, National Clinical Research Center for Respiratory Disease, National Center for Respiratory Medicine, Joint International Research Laboratory of Respiratory Health, Guangdong Basic Research Center of Excellence for Respiratory Medicine, Department of Respiratory Medicine, Guangzhou Institute of Respiratory Health, the First Affiliated Hospital of Guangzhou Medical University, Guangzhou, 510120 China; 2 Nanshan School, Guangzhou Medical University, Guangzhou, Guangdong, 511436 China; 3 Hetao Institute of Guangzhou National Laboratory, Shenzhen, 518000 China

**Keywords:** Chronic obstructive pulmonary disease, Sputum galectin-7, Clinical characteristics, Prognosis, Biomarker

## Abstract

**Objective:**

Galectin-7, a β-galactoside-binding lectin involved in inflammation, immune regulation, and apoptosis, has been implicated in the development and progression of various lung diseases. Inflammation, immune dysregulation, and epithelial cell apoptosis are key pathophysiological mechanisms underlying chronic obstructive pulmonary disease (COPD). However, the clinical significance of galectin-7 in COPD remains unclear. This study aimed to investigate the association between sputum galectin-7 levels and the clinical characteristics and prognosis of patients with COPD.

**Methods:**

In this prospective study, 150 patients with stable COPD and 50 healthy controls were enrolled. Demographic data, clinical parameters, and sputum samples were collected. Baseline galectin-7 concentrations in sputum supernatants were measured using an enzyme-linked immunosorbent assay (ELISA). Patients with COPD were followed for one year to document acute exacerbation events. Among COPD patients, Spearman correlation analysis and multivariable linear regression were performed to examine the associations between galectin-7 levels and clinical parameters or inflammatory markers, adjusting for sex, age, smoking status, and body mass index (BMI). Multivariable Poisson regression and logistic regression models were further applied to assess the relationship between galectin-7 levels and acute exacerbations within one year, with adjustment for the number of exacerbations in the previous year, sex, age, smoking status, BMI, and post-bronchodilator FEV1% predicted (Post-BD FEV1% pred).

**Results:**

After adjustment in the analysis of covariance, galectin-7 levels were significantly lower in patients with GOLD III–IV than in healthy controls and patients with GOLD I–II (P < 0.05). Multivariable linear regression analyses indicated that galectin-7 levels were significantly associated with multiple clinical indicators and inflammatory markers. In the multivariable Poisson regression model, each 10 ng/mL increase in sputum galectin-7 was associated with a 15.7% reduction in the risk of acute exacerbations within one year (RR, 0.843; 95% CI, 0.716–0.973; P = 0.028). In the multivariable logistic regression model, each 10 ng/mL increase in sputum galectin-7 was associated with a 35.1% lower risk of frequent acute exacerbations within one year (OR, 0.649; 95% CI, 0.424–0.923; P = 0.029).

**Conclusion:**

Lower sputum galectin-7 levels are associated with later stages and an increased risk of future acute exacerbations in COPD, suggesting that galectin-7 may be a promising biomarker related to disease severity and prognosis.

**Trial Registration:**

This study was registered in the International Clinical Trials Registry (NCT03240315). Trial registration on July 31, 2017.

**Supplementary Information:**

The online version contains supplementary material available at 10.1186/s12890-026-04171-9.

## Background

Chronic obstructive pulmonary disease (COPD) is a common chronic respiratory disorder characterized by persistent respiratory symptoms and irreversible airflow limitation [1]. It is currently the fourth leading cause of death worldwide, and the World Health Organization predicts that it will rise to the third leading cause of death by 2030 [2]. Acute exacerbations of COPD (AECOPD) are critical events in the disease course because they markedly accelerate the decline in lung function, increase mortality risk, impair quality of life, and raise healthcare costs [3]. A history of prior acute exacerbations is widely recognized as the strongest predictor of future events [4]. However, growing evidence indicates that additional clinical factors, including symptom burden, the degree of airflow obstruction, comorbidities, and inflammatory biomarkers, also help predict the risk of subsequent acute exacerbations [4–7]. As a noninvasive assessment method, sputum analysis provides a valuable approach for evaluating airway inflammation and identifying predictive biomarkers [8].

Galectin-7 is a galactose-binding protein predominantly expressed in epithelial cells and is widely involved in various physiological and pathological processes, including cell proliferation, migration, apoptosis, inflammation, and immune regulation [9]. As a bidirectional immune modulator, galectin-7 exerts distinct effects in different tissues and disease settings, where it may either promote or suppress disease progression through immunoregulatory pathways [10]. In recent years, an increasing number of studies have shown that members of the Galectin family are closely associated with the development and progression of many lung diseases [11–14]. Among these proteins, galectin-7 has gradually gained more scientific attention. Previous studies have reported aberrant expression of galectin-7 in diseases such as lung cancer and asthma, where it participates in key biological processes, including pulmonary inflammation, epithelial apoptosis, tissue remodeling, and immune microenvironment regulation, which collectively influence disease onset and progression [15–18]. Inflammation, immune dysregulation, and epithelial apoptosis are central pathophysiological mechanisms of COPD; however, no systematic research has examined the changes in galectin-7 expression in COPD or its clinical relevance.

This prospective cohort study aims to describe the levels of galectin-7 in the sputum of healthy subjects and patients with COPD of varying severity, and to assess its association with clinical characteristics and future disease exacerbations, thereby providing evidence for its potential application as a biomarker related to COPD severity and prognosis.

## Methods

### Study design

This prospective cohort study enrolled participants from the First Affiliated Hospital of Guangzhou Medical University between 2017 and 2019 (Clinical Trial Registration: NCT03240315). All participants provided written informed consent before enrollment, and the study was conducted in accordance with the ethical principles of the Declaration of Helsinki.

### Study population

Patients with COPD were recruited from the outpatient department of respiratory and critical care medicine. The inclusion criteria were age 40 to 90 years, a diagnosis of COPD based on the Global Initiative for Global Initiative for Chronic Obstructive Lung Disease (GOLD) guidelines (post-BD FEV_1_/FVC < 0.70), clinical stability defined as no exacerbations or additional treatments within the previous four weeks, and the ability to provide informed consent. Exclusion criteria included severe pulmonary diseases other than COPD (such as severe bronchiectasis, severe pneumonia, interstitial pneumonia, or lung cancer), autoimmune diseases, prior lung surgery, current systemic glucocorticoid therapy, and inability to complete pulmonary function testing or chest CT scans. Healthy subjects were recruited from a health examination center. Inclusion criteria for the healthy group were age 40 to 90 years, pre/post-bronchodilator FEV_1_/FVC ≥ 0.70, no history of lung disease, and no acute illness or medication use within the past month.

### Data collection

Demographic and clinical information was obtained from all participants. Chest CT scans, pulmonary function tests, and sputum and blood samples were collected, and the modified Medical Research Council (mMRC) scale [19] and COPD Assessment Test (CAT) [20] were administered. Patients with COPD were followed for one year. Acute exacerbations were assessed using two approaches: telephone follow-ups at three and nine months, and face-to-face assessments at six and twelve months. COPD severity was classified using GOLD spirometric grades based on post-BD FEV1% pred: mild (GOLD I, ≥ 80%), moderate (50% ≤GOLD II <80%), severe (30% ≤GOLD III <50%), and very severe (GOLD IV, < 30%) [21]. Acute exacerbations were defined as acute worsening of respiratory symptoms, including increased dyspnea, sputum volume, and sputum purulence, requiring extra medical visits or additional medication [22]. Frequent acute exacerbations were defined as two or more events within one year [23].

### Sputum collection

Sputum samples were obtained from all participants either by spontaneous expectoration or, when unsuccessful, by induction with hypertonic saline. The induction was performed using an ultrasonic nebulizer (WH-802, Guangdong Yuehua Medical Instrument Co., Ltd.) for 8 min. Participants initially inhaled 5% saline. If this concentration was not tolerated, it was replaced with a lower concentration (1%), and the process was repeated. Detailed protocols for sample collection and processing have been described previously [24].

### Measurement of IL family, MMP family inflammatory Factors, and galectin-7

Baseline sputum supernatant inflammatory factors were quantitatively measured using the multifactor protein chip Quantibody^®^ Array Glass Chip (QAH-CAA-6000, RayBiotech, Guangzhou, China), based on multiplex sandwich ELISA technology. These factors included the interleukin (IL) family (IL-1a, IL-1b, IL-1ra, IL-2, IL-2Ra, IL-2Rb, IL-5, IL-6, IL-6R, IL-7, IL-8, IL-10, IL-13, IL-16, IL-17 A, IL-17B, IL-17 F, IL-18BPa, and IL-28 A), the MMP family (MMP-1, MMP-8, MMP-9, and MMP-13), and galectin-7.

The assay procedure was as follows. First, the QAH-CAA-6000 slide chip was equilibrated at room temperature for 20 to 30 min, unsealed, and dried in a vacuum desiccator or at room temperature for one hour to ensure complete drying. Next, standards were prepared: 500 µL of sample dilution buffer was added to the cytokine standard mixture, followed by brief centrifugation and thorough mixing to generate Std 1; six clean centrifuge tubes were prepared and labeled as Std 2 to Std 7, and 200 µL of sample dilution buffer was added to each; a serial dilution was performed by transferring 100 µL from Std 1 to Std 2, mixing thoroughly, then transferring 100 µL from Std 2 to Std 3, and so on through Std 7. Simultaneously, a negative control (CNTRL) was prepared by adding 100 µL of sample dilution buffer into an additional clean tube. The detailed concentration gradients of each factor in the standard solutions are provided in Supplementary Material 1. Subsequently, 100 µL of sample dilution buffer was added to each well and incubated on a shaker at room temperature for one hour to block the antibody chip. After removal of the blocking solution, 60 µL of standard or test sample was added to each well and incubated overnight on a shaker at 4 °C. Washing was performed using a Thermo Scientific Wellwash Versa chip washer, repeated three times. For detection antibody incubation, the detection antibody mixture was centrifuged, reconstituted in 1.4 mL of sample dilution buffer, mixed thoroughly, and centrifuged again; 80 µL was added to each well and incubated on a shaker at room temperature for two hours, followed by repeated washing. For Cy3-streptavidin incubation, Cy3-streptavidin was centrifuged, reconstituted in 1.4 mL of sample dilution buffer, mixed thoroughly, and centrifuged again; 80 µL was added to each well, and the slide was covered with aluminum foil to protect it from light, then incubated on a shaker at room temperature for one hour, followed by the same washing steps. Finally, for fluorescence detection, the slide frame was removed, and signal acquisition was performed using the InnoScan 300 Microarray Scanner (Innopsys, France) with an excitation wavelength of 532 nm, a resolution of 10 μm, using the Cy3 green channel. Data were exported using the dedicated software provided with the QAH-CAA-6000.

Based on the tertiles of sputum galectin-7 concentration in COPD patients, participants were divided into three groups: the low galectin-7 group (< 4619.11 pg/mL), the moderate galectin-7 group (4619.11–15318.86 pg/mL), and the high galectin-7 group (> 15318.86 pg/mL).

### CT scan protocol

All subjects received deep breathing training prior to high-resolution CT (HRCT) scans. The scans were performed on a 64-slice spiral CT scanner (GE, Perspective-64) using the following parameters: tube voltage 110 kV, tube current automatically adjusted to about 30 mAs, pitch 1.3, matrix size 512 × 512, field of view 400 mm, reconstruction slice thickness and slice spacing both at 1.0 mm. Emphysema was defined as all lung voxels with a CT value below − 950 HU (Hounsfield units). Emphysema index (EI) was defined as the percentage of emphysematous volume to total lung volume. In patients with COPD, emphysema was defined as an emphysema index ≥ 5%, while those with an emphysema index < 5% were classified as the no emphysema group, and emphysema in chest CT images was quantitatively analyzed using VIDA software (Vida Diagnostics) [25].

### Statistical analysis

Statistical analyses were conducted using R software version 4.4.1. Unless otherwise specified, quantitative variables were described as mean ± standard deviation or median (interquartile range) according to their distribution normality, and categorical variables were presented as frequency and percentage. Participants with missing data were excluded from the analyses.

Sputum galectin-7 levels were compared among healthy controls, GOLD I, GOLD II, and GOLD III-IV groups using ANCOVA with covariates including age, sex, smoking status, smoking intensity and BMI. Quantitative variables between two groups were compared using an independent samples t-test or Mann-Whitney U test, while comparisons among three groups were performed using one-way ANOVA or Kruskal-Wallis H test. Categorical variables were compared using the chi-square test or Fisher’s exact test. Receiver operating characteristic (ROC) curve analysis was performed to evaluate the discriminative ability of sputum galectin-7. Specifically, ROC analyses were conducted to compare (1) healthy controls with patients at different stages of COPD; (2) patients with mild to moderate COPD (GOLD stages I–II) versus those with severe to very severe COPD (GOLD stages III–IV); and (3) the ability of galectin-7 to identify patients who developed future acute exacerbations or frequent acute exacerbations. Differences between ROC curves were compared using the DeLong test. Multivariable linear regression was employed to evaluate the relationships between galectin-7 levels and inflammatory factors or lung function, adjusted for sex, age, smoking status, and BMI. For linear regression between factors, we performed a log2 transformation of factor expression levels [26]. Multivariable Poisson and logistic regression analyses were applied to examine the association between galectin-7 levels and future acute exacerbations within one year, adjusted for exacerbations in the previous year, sex, age, smoking status, BMI, and Post-BD FEV_1_% pred.

## Results

### Patient characteristics

This study included a total of 200 participants, comprising 50 healthy controls, 21 patients with GOLD stage I, 60 with GOLD stage II, and 69 with GOLD stage III–IV (Table 1). Compared with healthy controls, patients with COPD were predominantly male, and the proportion of male participants increased progressively with advancing GOLD stages (68.0% in controls, 81.0% in GOLD stage I, 95.0% in GOLD stage II, and 97.1% in GOLD stage III–IV; *P* < 0.001). In addition, the mean age was significantly higher in COPD patients than in controls (58.7 ± 9.9 years in controls, 66.7 ± 7.6 years in GOLD stage I, 68.3 ± 9.2 years in GOLD stage II, and 65.8 ± 7.7 years in GOLD stage III–IV; *P* < 0.001). Smoking exposure increased with disease severity, with a median cumulative smoking exposure of 40.0 pack-years in patients with GOLD stage III–IV COPD (*P* < 0.001). Furthermore, body mass index (BMI), FEV_1_, and indices of small airway function (MMEF, MEF50, and MEF25) showed a gradual decline with increasing disease severity.

**Table 1:** Demographic, Clinical, and sputum biomarker profiles of 200 Participants, including healthy controls and COPD patients stratified by disease severity

Variable	Healthy	GOLD I	GOLD II	GOLD III–IV	*p*
n	50	21	60	69	
Clinical characteristics					
Gender, male(%)	34 (68.0)	17 (81.0)	57 (95.0)	67 (97.1)	**< 0.001**
Age, year (mean (SD))	58.74 (9.93)	66.67 (7.59)	68.25 (9.15)	65.83 (7.72)	**< 0.001**
Smoking history (%)					**< 0.001**
Current smoker	20 (40.0)	6 (28.6)	18 (30.0)	19 (27.5)	
Former smoker	10 (20.0)	8 (38.1)	33 (55.0)	48 (69.6)	
Never smoker	20 (40.0)	7 (33.3)	9 (15.0)	2 (2.9)	
Smoking intensity, pack/years (median [IQR])	7.50 [0.00, 30.00]	27.50 [0.00, 40.00]	26.00 [15.00, 42.50]	40.00 [27.75, 51.00]	**< 0.001**
Height, cm (mean (SD))	161.50 (7.57)	160.79 (7.11)	164.88 (6.16)	164.87 (5.92)	**0.004**
Weight, kg (mean (SD))	64.92 (10.72)	59.38 (9.33)	63.87 (9.27)	56.62 (10.37)	**< 0.001**
BMI, kg/m2 (median [IQR])	23.94 [22.53, 26.71]	22.65 [20.56, 24.77]	23.72 [21.99, 25.14]	20.90 [18.07, 22.96]	**< 0.001**
Lung physiology characteristics					
Pre-BD FEV_1_/FVC (median [IQR])	0.77 [0.73, 0.81]	0.63 [0.58, 0.66]	0.54 [0.47, 0.58]	0.34 [0.31, 0.41]	**< 0.001**
Pre-BD FEV_1_, L (mean (SD))	2.65 (0.63)	2.16 (0.52)	1.57 (0.35)	0.86 (0.22)	**< 0.001**
Pre-BD FEV_1_% pred (mean (SD))	101.04 (13.31)	88.62 (7.66)	60.48 (9.30)	32.36 (7.23)	**< 0.001**
Pre-BD FVC (L) (median [IQR])	3.44 [2.75, 4.00]	3.58 [3.16, 3.92]	2.88 [2.60, 3.38]	2.34 [2.08, 2.80]	**< 0.001**
Pre-BD PEF (mean (SD))	7.81 (1.90)	6.00 (1.91)	4.63 (1.39)	2.50 (0.83)	**< 0.001**
Pre-BD MMEF (median [IQR])	2.09 [1.49, 2.61]	0.92 [0.74, 1.16]	0.54 [0.40, 0.68]	0.24 [0.20, 0.29]	**< 0.001**
Pre-BD MEF50 (median [IQR])	2.78 [2.15, 3.38]	1.36 [1.10, 1.62]	0.72 [0.58, 0.94]	0.29 [0.23, 0.33]	**< 0.001**
Pre-BD MEF25 (median [IQR])	0.69 [0.50, 0.91]	0.30 [0.27, 0.41]	0.20 [0.16, 0.25]	0.13 [0.10, 0.15]	**< 0.001**
Sputum					
galectin-7,pg/ml (median [IQR])	24239.56 [13460.16, 36441.39]	14275.12 [7880.17, 20004.93]	15318.86 [8120.00, 24239.56]	3899.17 [1346.43, 13460.16]	**< 0.001**
IL-1a, pg/ml (median [IQR])	58.53 [12.80, 148.85]	12.02 [6.00, 29.99]	31.34 [7.64, 75.68]	62.89 [15.28, 122.32]	**0.032**
IL-1b, pg/ml (median [IQR])	31.34 [16.21, 66.71]	17.09 [8.64, 32.68]	29.99 [11.69, 67.98]	85.98 [27.78, 148.85]	**< 0.001**
IL-1ra, pg/ml (median [IQR])	202.01 [122.32, 327.29]	148.85 [122.32, 255.20]	190.30 [122.32, 258.56]	148.85 [98.49, 283.69]	0.700
IL-2 Ra, pg/ml (median [IQR])	28.89 [0.87, 113.24]	5.58 [0.74, 35.46]	1.93 [0.74, 18.96]	2.39 [0.58, 114.71]	0.301
IL-2 Rb, pg/ml (median [IQR])	1833.82 [1069.60, 3317.61]	2179.95 [1620.09, 3317.61]	2501.66 [1290.16, 3523.60]	2276.79 [1410.73, 3064.58]	0.762
IL-5,pg/ml (median [IQR])	1.59 [0.68, 3.95]	2.39 [1.03, 6.91]	2.28 [0.89, 10.73]	8.64 [3.30, 23.07]	**< 0.001**
IL-6,pg/ml (median [IQR])	74.22 [47.28, 222.33]	139.64 [91.66, 215.32]	202.01 [96.78, 419.59]	432.85 [168.50, 822.53]	**< 0.001**
IL-6R, pg/ml (median [IQR])	455.13 [375.71, 617.27]	393.82 [331.99, 527.49]	477.42 [393.82, 719.03]	527.49 [415.17, 822.53]	**0.024**
IL-7,pg/ml (median [IQR])	5.16 [3.40, 10.73]	5.16 [2.94, 9.55]	5.58 [2.10, 19.45]	7.64 [3.30, 20.78]	0.438
IL-8,pg/ml (median [IQR])	283.69 [190.30, 454.17]	313.19 [202.01, 527.49]	331.99 [229.11, 542.47]	331.99 [202.01, 647.19]	0.304
IL-10,pg/ml (median [IQR])	2.39 [0.82, 6.00]	2.94 [1.59, 6.00]	7.28 [1.51, 27.78]	12.02 [5.16, 35.46]	**< 0.001**
IL-13,pg/ml (median [IQR])	1.59 [0.66, 3.95]	1.59 [0.74, 4.78]	1.93 [0.64, 10.73]	2.16 [0.74, 6.91]	0.440
IL-16,pg/ml (median [IQR])	229.11 [100.45, 428.43]	298.20 [168.50, 503.75]	372.84 [130.93, 625.31]	587.39 [375.71, 944.38]	**< 0.001**
IL-17 A, pg/ml (median [IQR])	5.58 [2.05, 14.85]	6.00 [1.93, 12.02]	8.14 [3.67, 23.64]	13.58 [8.64, 46.35]	**< 0.001**
IL-17B, pg/ml (median [IQR])	1262.01 [858.95, 2135.21]	1682.64 [858.95, 2730.52]	900.31 [514.97, 1875.62]	527.49 [351.85, 944.38]	**< 0.001**
IL-17 F, pg/ml (median [IQR])	1.93 [0.38, 29.26]	190.30 [12.02, 503.75]	127.24 [1.89, 535.04]	20.78 [1.03, 503.75]	**< 0.001**
IL-18 BPa, pg/ml (median [IQR])	3.12 [0.47, 50.07]	12.02 [0.51, 215.32]	58.53 [1.72, 298.20]	9.10 [1.03, 91.66]	**0.013**
IL-28 A, pg/ml (median [IQR])	1.33 [0.38, 5.06]	6.00 [0.74, 27.78]	5.81 [1.25, 20.02]	4.41 [1.03, 12.02]	**0.015**
MMP-1,pg/ml (median [IQR])	572.54 [5.75, 1152.08]	557.69 [91.66, 1346.43]	988.99 [428.43, 2867.98]	2087.34 [527.49, 4082.01]	**< 0.001**
MMP-8,pg/ml (median [IQR])	8635.63 [7605.50, 10490.74]	8242.27 [6866.60, 10030.44]	9101.80 [7513.94, 10490.74]	10490.74 [8635.63, 11844.25]	**< 0.001**
MMP-9,pg/ml (median [IQR])	10812.03 [7880.17, 13460.16]	7880.17 [5211.81, 10490.74]	9786.39 [7431.13, 13663.90]	14275.12 [10030.44, 18485.60]	**< 0.001**
MMP-13,pg/ml (median [IQR])	185.03 [48.31, 477.42]	393.82 [98.49, 618.01]	375.71 [190.30, 841.97]	298.20 [122.32, 743.14]	**0.012**

Data are presented as No. (%), median (interquartile range), mean [SD], or as otherwise indicated

*COPD* Chronic Obstructive Pulmonary Disease, *Pre-BD* Pre-Bronchodilator, *FEV*_1_ Forced Expiratory Volume in 1 s, *FVC* Forced Vital Capacity, *PEF* Peak Expiratory Flow, *MMEF* Maximal Mid-Expiratory Flow, *MEF50* Maximal Expiratory Flow at 50% of Forced Vital Capacity, *MEF25* Maximal Expiratory Flow at 25% of Forced Vital Capacity, *BMI* Body Mass Index, *FEV*_1_*% pred* Forced Expiratory Volume in 1 s (Actual/Predicted), *galectin-7* Galactoside-binding lectin-7, *IL-1a * Interleukin-1 alpha, *IL-1b * Interleukin-1 beta, *IL-1ra* Interleukin-1 receptor antagonist, *IL-2Ra* Interleukin-2 receptor alpha, *IL-2Rb* Interleukin-2 receptor beta, *IL-5* Interleukin-5, *IL-6* Interleukin-6, *IL-6R* Interleukin-6 receptor, *IL-7* Interleukin-7, *IL-8* Interleukin-8, *IL-10* Interleukin-10, *IL-13* Interleukin-13, *IL-16* Interleukin-16, *IL-17 A* Interleukin-17 A, *IL-17B* Interleukin-17B, *IL-17 F* Interleukin-17 F, *IL-18BPa* Interleukin-18 binding protein alpha, *IL-28 A* Interleukin-28 A, *MMP-1* Matrix Metalloproteinase-1, *MMP-8* Matrix Metalloproteinase-8, *MMP-9* Matrix Metalloproteinase-9, *MMP-13* Matrix Metalloproteinase-13

*P*-values < 0.05 are shown in boldface

**Fig. 1:**
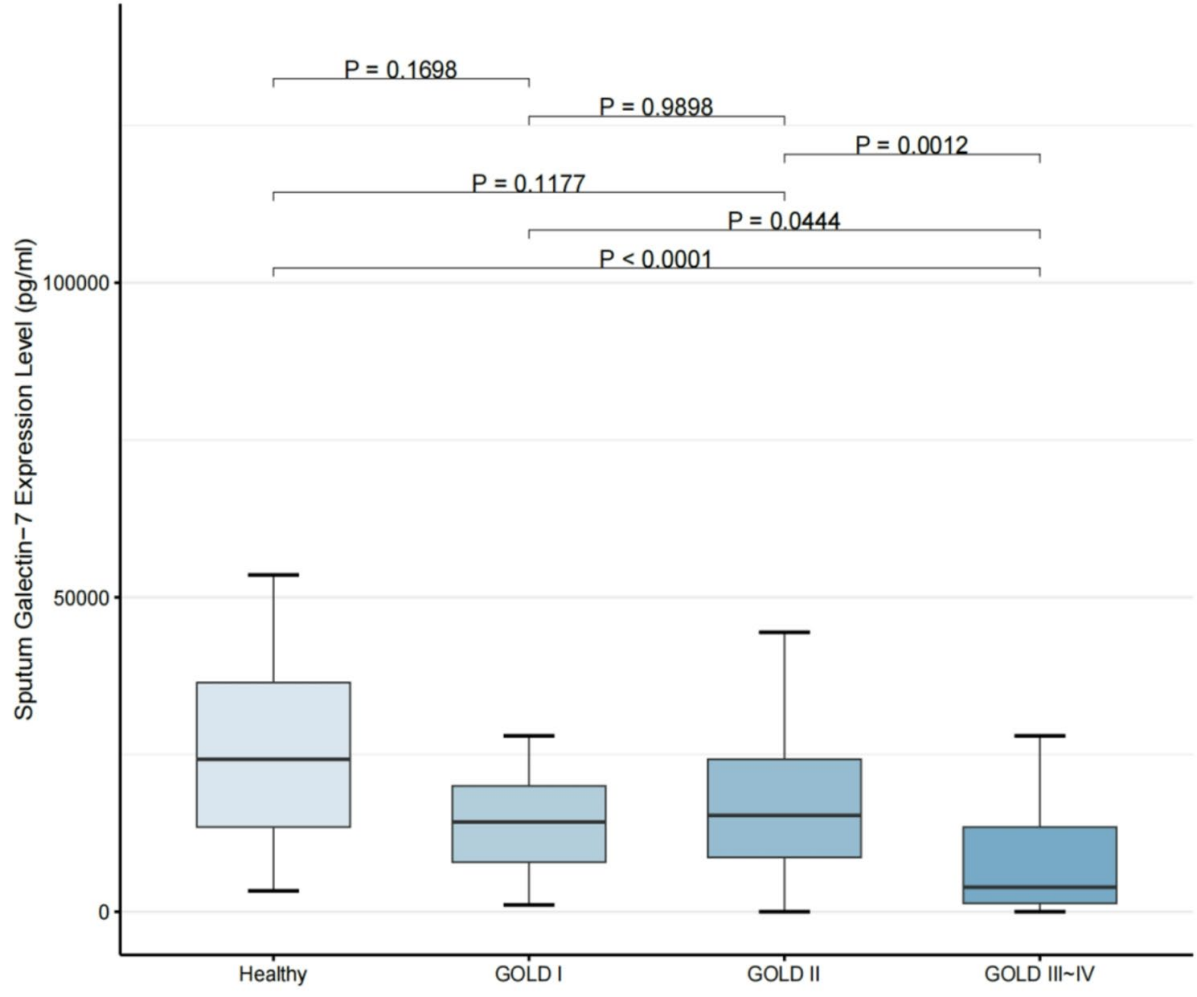
Analysis of covariance (ANCOVA) of sputum galectin-7 levels among different groups (Healthy, GOLD I, GOLD II, GOLD III–IV), adjusted for age, sex, smoking status, smoking intensity and BMI

Figure 1 illustrates sputum galectin-7 expression levels in samples obtained from healthy subjects and patients across different GOLD stages. No significant differences were observed between healthy controls and patients with GOLD stage I or II disease, nor between GOLD stage I and stage II (all *P* > 0.05). In contrast, compared with patients in the GOLD III–IV group, healthy controls (*P* < 0.001) as well as patients with GOLD stage I (*P* = 0.0444) and GOLD stage II (*P* = 0.001) exhibited significantly higher levels of sputum galectin-7.

The demographic and clinical characteristics of COPD participants stratified by galectin-7 levels are summarized in Table 2. No statistically significant differences were observed among the groups in sex distribution, age, smoking history, or medication history (*P* > 0.05). However, decreasing galectin-7 levels were accompanied by corresponding reductions in BMI, FEV_1_, FVC and small airway function, as well as a higher proportion of patients with severe COPD. Lower galectin-7 levels were also associated with elevated serum globulin, higher CAT and mMRC scores, and an increased emphysema index, with the low galectin-7 group demonstrating significantly greater emphysema burden compared with the moderate and high galectin-7 groups (*P* = 0.003). In addition, the proportion of frequent exacerbators increased significantly with decreasing galectin-7 levels (high galectin-7 group 16% vs. moderate galectin-7 group 34.8% vs. low galectin-7 group 38.3%, *P* = 0.034).

**Table 2:** Demographic and clinical characteristics of COPD patients stratified by galectin-7 levels

Variables	High galectin-7	Moderate galectin-7	Low galectin-7	*p*
**n**	50	50	50	
Clinical characteristics				
Gender, Male (%)	48 (96.0)	44 (88.0)	49 (98.0)	0.084
Age, Year (mean (SD))	67.54 (7.96)	66.48 (8.77)	66.72 (8.36)	0.802
Smoking history (%)				0.085
Current smoker	20 (40.0)	13 (26.0)	10 (20.0)	
Former smoker	25 (50.0)	28 (56.0)	36 (72.0)	
Never smoker	5 (10.0)	9 (18.0)	4 (8.0)	
Smoking intensity, pack /years (median [IQR])	30.20 [15.00, 49.50]	27.50 [10.00, 47.50]	37.50 [25.00, 50.00]	0.223
Height, cm (mean (SD))	164.34 (6.35)	164.59 (6.49)	163.98 (6.20)	0.890
Weight, kg (mean (SD))	63.71 (10.35)	60.35 (9.00)	55.66 (10.07)	**< 0.001**
BMI, kg/m^2^ (mean (SD))	23.53 (3.19)	22.27 (3.06)	20.69 (3.52)	**< 0.001**
Lung physiology characteristics				
GOLD Classification(%)				**0.001**
GOLD I	8 (16.0)	10 (20.0)	3 (6.0)	
GOLD II	28 (56.0)	22 (44.0)	10 (20.0)	
GOLD III	13 (26.0)	14 (28.0)	29 (58.0)	
GOLD IV	1 (2.0)	4 (8.0)	8 (16.0)	
Post-BD FEV_1_/FVC (median [IQR])	0.54 [0.44, 0.62]	0.50 [0.40, 0.59]	0.39 [0.33, 0.49]	**< 0.001**
Post-BD FEV_1_, L (median [IQR])	1.61 [1.20, 2.04]	1.56 [1.11, 1.85]	1.01 [0.85, 1.35]	**< 0.001**
Post-BD FEV_1_% pred (median [IQR])	62.79 [48.00, 73.61]	62.14 [43.12, 75.95]	39.87 [33.70, 50.67]	**< 0.001**
Post-BD FVC, L (median [IQR])	3.06 [2.56, 3.75]	3.00 [2.78, 3.59]	2.60 [2.34, 3.03]	**0.004**
Post-BD PEF (median [IQR])	4.90 [3.38, 6.02]	4.44 [3.42, 5.51]	2.78 [2.04, 3.78]	**< 0.001**
Post-BD MMEF (median [IQR])	0.60 [0.37, 0.87]	0.50 [0.34, 0.78]	0.31 [0.24, 0.42]	**< 0.001**
Post-BD MEF50 (median [IQR])	0.78 [0.46, 1.23]	0.70 [0.39, 1.01]	0.39 [0.28, 0.53]	**< 0.001**
Post-BD MEF25 (median [IQR])	0.23 [0.14, 0.30]	0.21 [0.15, 0.25]	0.15 [0.13, 0.20]	**0.009**
Questionnaire score				
CAT Score (mean (SD))	7.69 (4.01)	9.46 (5.34)	10.10 (4.90)	**0.038**
mMRC Score (mean (SD))	1.22 (0.90)	1.46 (0.91)	1.72 (1.01)	**0.035**
Medication use				
ICS, N (%)	31 (62.0)	31 (62.0)	35 (70.0)	0.627
LABA, N (%)	32 (64.0)	31 (62.0)	35 (70.0)	0.682
LAMA, N (%)	20 (40.0)	21 (42.0)	30 (60.0)	0.088
Laboratory examination index				
Blood WBC (median [IQR])	6.04 [5.35, 7.47]	6.50 [5.88, 7.46]	6.93 [5.86, 8.85]	0.058
Blood NEU (median [IQR])	3.80 [2.80, 4.20]	3.80 [3.20, 4.60]	4.25 [3.40, 5.27]	0.062
Blood LYM (median [IQR])	1.80 [1.30, 2.20]	1.85 [1.48, 2.30]	1.90 [1.33, 2.30]	0.469
Blood Monocytes (median [IQR])	0.50 [0.40, 0.60]	0.50 [0.40, 0.52]	0.46 [0.40, 0.60]	0.638
Blood EOS (median [IQR])	0.21 [0.12, 0.28]	0.20 [0.12, 0.37]	0.22 [0.13, 0.34]	0.710
Serum globulin (median [IQR])	30.40 [28.72, 31.90]	32.70 [31.13, 34.27]	31.80 [29.95, 35.30]	**< 0.001**
HsCRP (median [IQR])	0.77 [0.33, 1.79]	1.03 [0.37, 1.82]	1.66 [0.52, 5.00]	0.056
FeNO (median [IQR])	22.00 [14.50, 35.00]	25.00 [16.75, 36.00]	26.00 [17.50, 35.50]	0.660
CT scan				
Emphysema Index (median [IQR])	4.50 [2.08, 15.11]	4.85 [1.59, 16.90]	18.08 [6.81, 24.62]	**0.003**
History of exacerbations				
Exacerbations in the past 1 year (mean (SD))	0.73 (1.12)	0.58 (0.67)	1.14 (1.72)	0.071
Exacerbations in the following year (mean (SD))	0.72 (0.93)	1.11 (1.08)	1.23 (1.20)	0.051
frequent exacerbation, N (%)	8 (16.0)	16 (34.8)	18 (38.3)	**0.034**
Sputum				
galectin-7,pg/ml (median [IQR])	24239.56 [18485.60, 36441.39]	10812.03 [7265.50, 14071.38]	1318.30 [432.85, 2065.76]	**< 0.001**
IL-1a, pg/ml (median [IQR])	25.43 [7.64, 78.58]	25.43 [5.26, 78.58]	65.43 [21.63, 213.96]	**0.002**
IL-1b, pg/ml (median [IQR])	19.00 [10.73, 52.22]	25.34 [10.89, 74.22]	118.52 [50.07, 309.51]	**< 0.001**
IL-1ra, pg/ml (median [IQR])	169.10 [124.48, 255.20]	196.15 [98.49, 313.19]	139.64 [100.45, 242.07]	0.528
IL-2 Ra, pg/ml (median [IQR])	1.93 [0.78, 10.92]	2.41 [0.68, 29.99]	4.81 [0.51, 199.08]	0.442
IL-2 Rb, pg/ml (median [IQR])	2559.89 [1352.75, 3448.49]	2001.01 [1083.35, 3317.61]	2276.79 [1833.82, 3383.88]	0.412
IL-5,pg/ml (median [IQR])	2.41 [0.78, 10.43]	5.37 [1.21, 15.66]	8.37 [1.99, 26.89]	**0.021**
IL-6,pg/ml (median [IQR])	235.26 [118.76, 593.61]	196.15 [100.45, 399.34]	424.01 [171.32, 794.64]	**0.016**
IL-6R, pg/ml (median [IQR])	443.51 [375.71, 557.69]	465.80 [362.34, 610.36]	618.01 [432.85, 858.95]	**0.002**
IL-7,pg/ml (median [IQR])	7.64 [4.41, 20.33]	8.64 [4.41, 23.07]	3.86 [1.93, 12.02]	**0.047**
IL-8,pg/ml (median [IQR])	331.99 [229.11, 521.56]	393.82 [229.11, 579.97]	298.20 [190.30, 639.90]	0.668
IL-10,pg/ml (median [IQR])	6.91 [2.05, 19.86]	5.58 [1.68, 11.21]	23.07 [5.37, 53.15]	**< 0.001**
IL-13,pg/ml (median [IQR])	1.85 [0.68, 12.02]	2.16 [1.03, 6.13]	1.93 [0.58, 7.61]	0.787
IL-16,pg/ml (median [IQR])	315.09 [153.76, 572.42]	415.17 [140.32, 703.42]	647.19 [459.98, 944.38]	**0.003**
IL-17 A, pg/ml (median [IQR])	8.39 [3.12, 24.77]	10.73 [5.16, 20.78]	13.65 [8.64, 34.76]	**0.030**
IL-17B, pg/ml (median [IQR])	1099.21 [655.57, 1980.30]	944.38 [527.49, 1950.63]	424.01 [196.55, 632.24]	**< 0.001**
IL-17 F, pg/ml (median [IQR])	10.08 [1.80, 383.33]	185.03 [0.82, 557.69]	88.82 [1.59, 407.64]	0.718
IL-18 BPa, pg/ml (median [IQR])	10.08 [1.76, 114.71]	54.17 [1.06, 268.65]	16.08 [1.42, 130.93]	0.723
IL-28 A, pg/ml (median [IQR])	7.46 [1.76, 12.02]	4.97 [0.68, 26.61]	4.23 [1.42, 12.86]	0.950
MMP-1,pg/ml (median [IQR])	711.00 [317.89, 1939.57]	762.37 [270.29, 1986.16]	3135.45 [1512.75, 7352.10]	**< 0.001**
MMP-8,pg/ml (median [IQR])	8242.27 [7182.69, 10490.74]	9322.07 [8242.27, 10490.74]	10490.74 [9101.80, 11844.25]	**< 0.001**
MMP-9,pg/ml (median [IQR])	7697.06 [5534.75, 9908.41]	10490.74 [7970.70, 13460.16]	16885.54 [14275.12, 24239.56]	**< 0.001**
MMP-13,pg/ml (median [IQR])	261.92 [93.37, 595.38]	331.99 [91.66, 557.69]	557.44 [298.20, 1075.53]	**< 0.001**

Data are presented as No. (%), median (interquartile range), mean [SD], or as otherwise indicated

*GOLD* Global Initiative for Chronic Obstructive Lung Disease, *CAT* COPD Assessment Test, *mMRC* Modified Medical Research Council dyspnea scale, *ICS* Inhaled Corticosteroids, *LABA* Long-Acting Beta₂-Agonist, *LAMA* Long-Acting Muscarinic Antagonist, *WBC* White Blood Cell, *NEU* Neutrophil, *LYM* Lymphocyte, *Monocytes* Monocyte, *EOS* Eosinophil, *Serum globulin* Serum Globulin, *HsCRP* High-Sensitivity C-Reactive Protein, *FeNO* Fractional Exhaled Nitric Oxide, *Emphysema Index* Emphysema Index

*P*-values < 0.05 are shown in boldface

### The diagnostic and differential efficacy of sputum galectin-7 in COPD

**Fig. 2:**
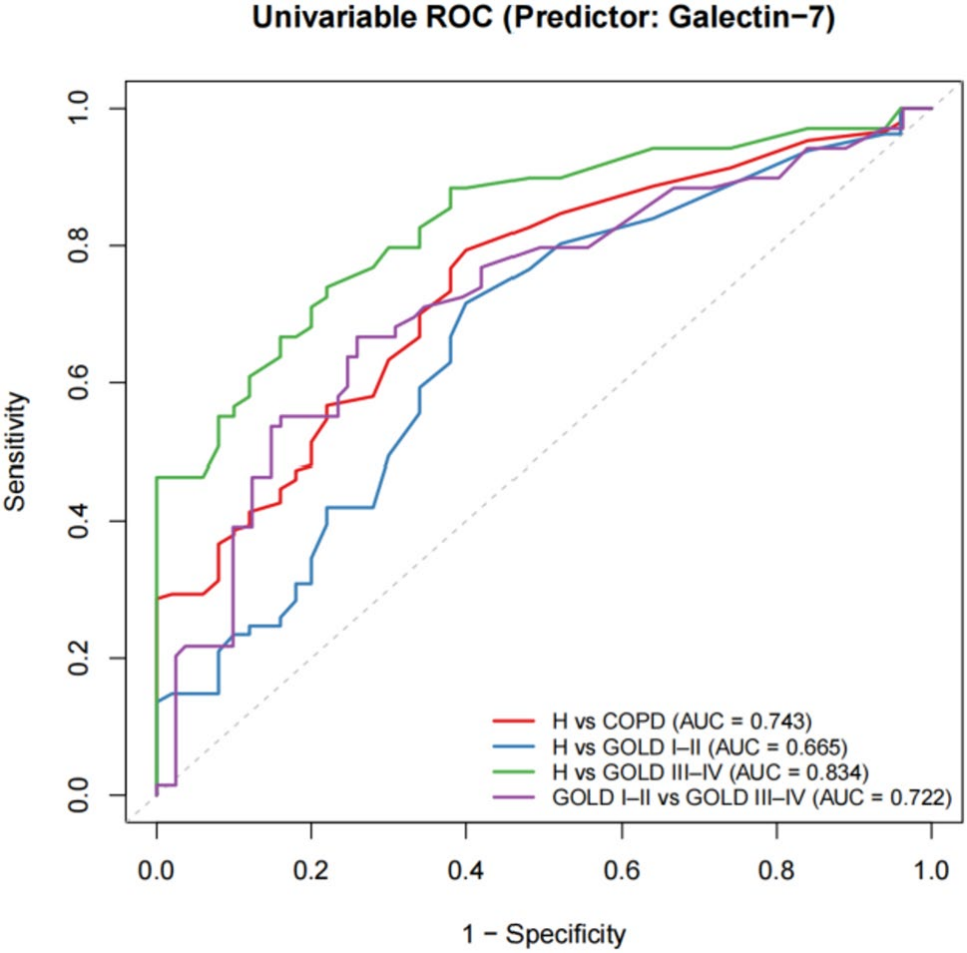
ROC curves of sputum galectin-7 for discriminating healthy controls from all COPD patients, healthy controls from GOLD I–II, healthy controls from GOLD III–IV, and GOLD I–II from GOLD III–IV

ROC curve analysis was performed to assess the discriminative ability of sputum galectin-7 in distinguishing healthy subjects from COPD patients and in differentiating COPD severity strata (Fig. 2). Galectin-7 demonstrated moderate discrimination between healthy subjects and all COPD patients, with an AUC of 0.743 (sensitivity 0.793, specificity 0.600). Stratified analyses revealed limited discrimination between healthy subjects and patients with GOLD I–II COPD (AUC 0.665; sensitivity 0.716, specificity 0.600), whereas discrimination was good between healthy subjects and those with GOLD III–IV COPD (AUC 0.834; sensitivity 0.739, specificity 0.780). Furthermore, galectin-7 was able to moderately differentiate between GOLD stages I–II and GOLD stages III–IV, with an AUC value of 0.722 (sensitivity of 0.667 and specificity of 0.741).

### Analysis between sputum galectin-7 and clinical variables

**Fig. 3:**
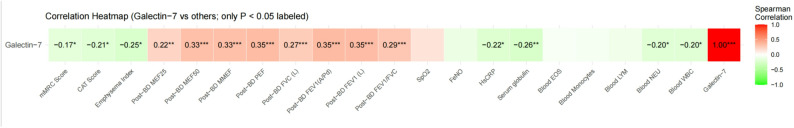
**Spearman correlation heatmap of galectin-7 and clinical indicators .** Red indicates positive correlations, and green indicates negative correlations, with color intensity reflecting correlation strength. Statistical significance is denoted as *P < 0.05, **P < 0.01, and ***P < 0.001. Only correlations with *P* < 0.05 are labeled

This study employed Spearman rank correlation analysis to investigate the relationships between sputum galectin-7 and various clinical indicators in COPD patients (Fig. 3). The results demonstrated significant correlations between galectin-7 and multiple clinical parameters, including Post-BD MEF25, Post-BD MEF50, Post-BD MMEF, Post-BD PEF, Post-BD FVC, Post-BD FEV_1_, Post-BD FEV_1_/FVC, Emphysema, HsCRP, serum globulin, blood NEU, blood WBC, mMRC score, and CAT score (*P* < 0.05).

To further evaluate the independent associations between sputum galectin-7 levels and clinical indicators, linear regression analyses were performed. Table 3 summarizes the clinical variables that remained statistically significant (*P* < 0.05) in the multivariable linear regression model, together with results from both univariable and multivariable analyses.The linear regression analysis demonstrated that lower sputum galectin-7 levels were independently associated with a higher inflammatory burden and worse clinical status in COPD patients. Specifically, for each 10 ng/mL decrease in sputum galectin-7, the CAT score increased by 0.589 points, while serum globulin, blood white blood cell count, and blood neutrophil count increased by 0.809 g/L, 0.297 × 10^9^/L, and 0.226 × 10^9^/L, respectively. In addition, reduced sputum galectin-7 levels were significantly associated with decreased post-bronchodilator lung function parameters, including FEV_1_, FVC, and PEF, as well as small airway indices (Post-BD MMEF and MEF50). (detailed results are presented in Table 3)

**Table 3:** Linear regression analysis of clinical variables for sputum galectin-7 levels

Variables(Y)	Univariate analysis			Multivariate analysis		
	β	95% CI	*P*	β	95% CI	*P*
CAT score	-0.764	(-1.337, -0.19)	**0.009**	-0.589	(-1.162, -0.016)	**0.044**
Post-BD FEV_1_	0.116	(0.054, 0.179)	**< 0.001**	0.086	(0.026, 0.146)	**0.005**
Post-BD FEV_1_%pred	3.987	(1.571, 6.404)	**0.001**	2.332	(0.031, 4.632)	**0.047**
Post-BD FVC	0.125	(0.048, 0.202)	**0.002**	0.135	(0.063, 0.207)	**< 0.001**
Post-BD PEF	0.346	(0.135, 0.557)	**0.001**	0.258	(0.055, 0.461)	**0.013**
Post-BD MMEF	0.065	(0.024, 0.105)	**0.002**	0.050	(0.009, 0.09)	**0.016**
Post-BD MEF50	0.097	(0.041, 0.154)	**< 0.001**	0.069	(0.014, 0.124)	**0.014**
Blood WBC	-0.297	(-0.519, -0.075)	**0.009**	-0.297	(-0.519, -0.075)	**0.009**
Blood NEU	-0.226	(-0.412, -0.04)	**0.018**	-0.226	(-0.412, -0.04)	**0.018**
Serum globulin	-0.788	(-1.232, -0.344)	**< 0.001**	-0.809	(-1.245, -0.373)	**< 0.001**

Multivariate linear regression analysis was adjusted for age, gender, smoking status, and BMI. β, Regression Coefficient; 95% CI, 95% confidence interval

*P*-values < 0.05 are shown in boldface

### Analysis of galectin-7 and cytokines in sputum

Linear regression analysis was performed to evaluate the associations between sputum galectin-7 and these interleukins and matrix metalloproteinases in COPD patients. Table 4 presents the factor variables with *p* < 0.05 in the multivariable linear analysis of sputum galectin-7 levels, along with results from both univariable and multivariable linear regression analyses for these significant variables. The analysis demonstrated that for each 10 ng/mL increase in sputum galectin-7, significant changes occurred in multiple inflammatory factors and matrix metalloproteinases (*P* < 0.05). Specifically, MMP-1, MMP-8, MMP-9, and MMP-13 from the MMP family and IL-1a, IL-1b, IL-2Ra, IL-6R, IL-8, IL-10, and IL-17 A from the interleukin family showed significant decreases, whereas IL-17B showed significant increases.

**Table 4:** Linear regression analysis of cytokines and MMPs for sputum galectin-7 levels

Variables(Y)	Univariate analysis			Multivariate analysis		
	β	95% CI	*P*	β	95% CI	*P*
IL-1a	-0.260	(-0.383, -0.136)	**< 0.001**	-0.234	(-0.366, -0.102)	**< 0.001**
IL-1b	-0.398	(-0.494, -0.302)	**< 0.001**	-0.402	(-0.504, -0.299)	**< 0.001**
IL-2 Ra	-0.235	(-0.396, -0.074)	**0.005**	-0.235	(-0.396, -0.074)	**0.005**
IL-6R	-0.079	(-0.115, -0.043)	**< 0.001**	-0.079	(-0.115, -0.043)	**< 0.001**
IL-8	-0.053	(-0.106, 0.001)	**0.054**	-0.062	(-0.115, -0.01)	**0.02**
IL-10	-0.290	(-0.383, -0.197)	**< 0.001**	-0.260	(-0.358, -0.162)	**< 0.001**
IL-17 A	-0.155	(-0.246, -0.064)	**< 0.001**	-0.141	(-0.234, -0.048)	**0.003**
IL-17B	0.395	(0.307, 0.484)	**< 0.001**	0.395	(0.307, 0.484)	**< 0.001**
MMP-1	-0.270	(-0.45, -0.089)	**0.004**	-0.250	(-0.431, -0.068)	**0.007**
MMP-8	-0.033	(-0.055, -0.012)	**0.003**	-0.025	(-0.048, -0.002)	**0.036**
MMP-9	-0.183	(-0.221, -0.145)	**< 0.001**	-0.180	(-0.221, -0.139)	**< 0.001**
MMP-13	-0.312	(-0.445, -0.179)	**< 0.001**	-0.312	(-0.445, -0.179)	**< 0.001**

Multivariate linear regression analysis was adjusted for age, gender, smoking status, and BMI. β,Regression Coefficient; 95% CI, 95% confidence interval

*P*-values < 0.05 are shown in boldface

### Association between sputum galectin-7 levels and emphysema severity

**Fig. 4:**
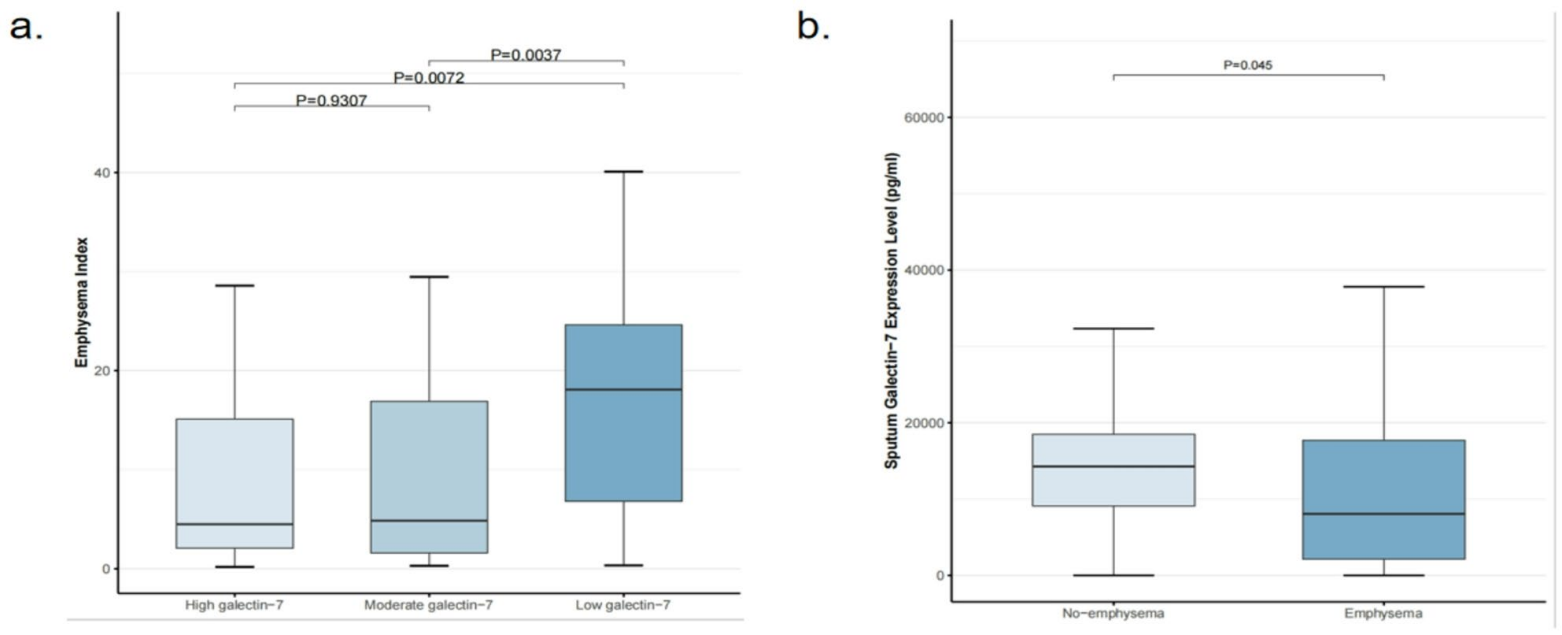
Bidirectional comparison between emphysema and sputum galectin-7: (**a**) Emphysema Index in high, moderate, and low sputum galectin-7 groups; (**b**) Sputum galectin-7 levels (pg/mL) in participants with and without emphysema

In this study, we observed a negative correlation between emphysema and sputum galectin-7 levels (*R*= -0.25, *P* < 0.05, Fig. 3); subsequently, COPD patients were categorized into high, moderate, and low galectin-7 groups based on their sputum galectin-7 expression levels, and the emphysema indices among the three groups were compared (Fig. 4,a). Compared with the low galectin-7 group (18.08 [6.81, 24.62]), both the high galectin-7 group (4.85 [1.59, 19.90]; *P* = 0.007) and the moderate galectin-7 group (4.50 [2.08, 15.11]; *P* = 0.003) exhibited significantly lower emphysema indices. Furthermore, we investigated the differences in galectin-7 expression between COPD patients with and without emphysema, and the results showed that galectin-7 levels were significantly lower in COPD patients with emphysema compared to those without emphysema (8061.22 [2135.21, 17700.99] vs. 14275.12 [9088.98, 18485.60], *P* = 0.045; Fig. 4, b).

### Association analysis of sputum galectin-7 with acute exacerbations of COPD

**Fig. 5:**
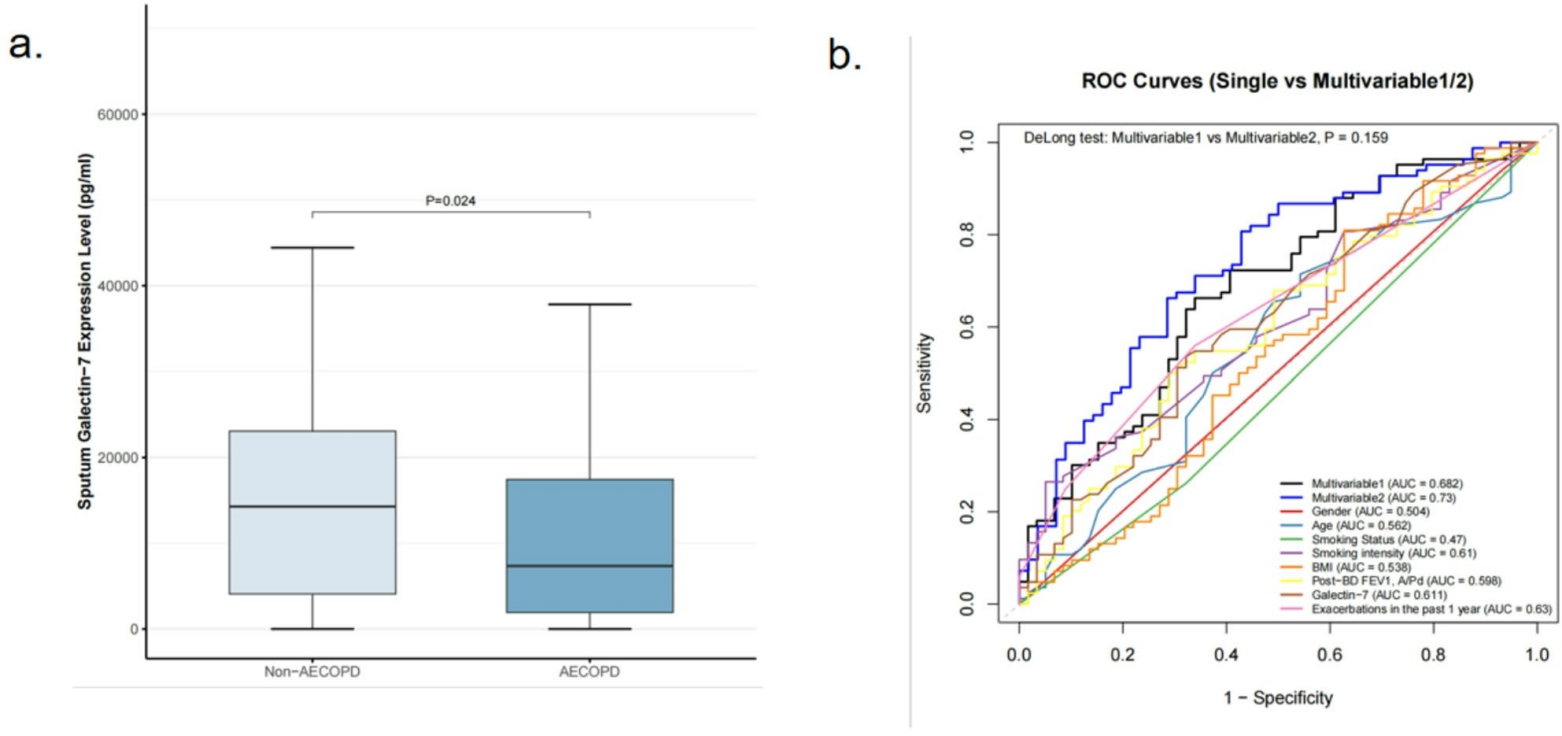
Sputum galectin-7 and future acute exacerbations in COPD: (**a**) comparison of baseline sputum galectin-7 levels between AECOPD and non-AECOPD groups; (**b**) prediction of future acute exacerbations using age, sex, smoking status, BMI, lung function, and other confounders, exacerbation history, and sputum galectin-7, with performance comparison between Multivariable Model 1 (confounders + galectin-7) and Multivariable Model 2 (confounders + exacerbation history)

**Fig. 6:**
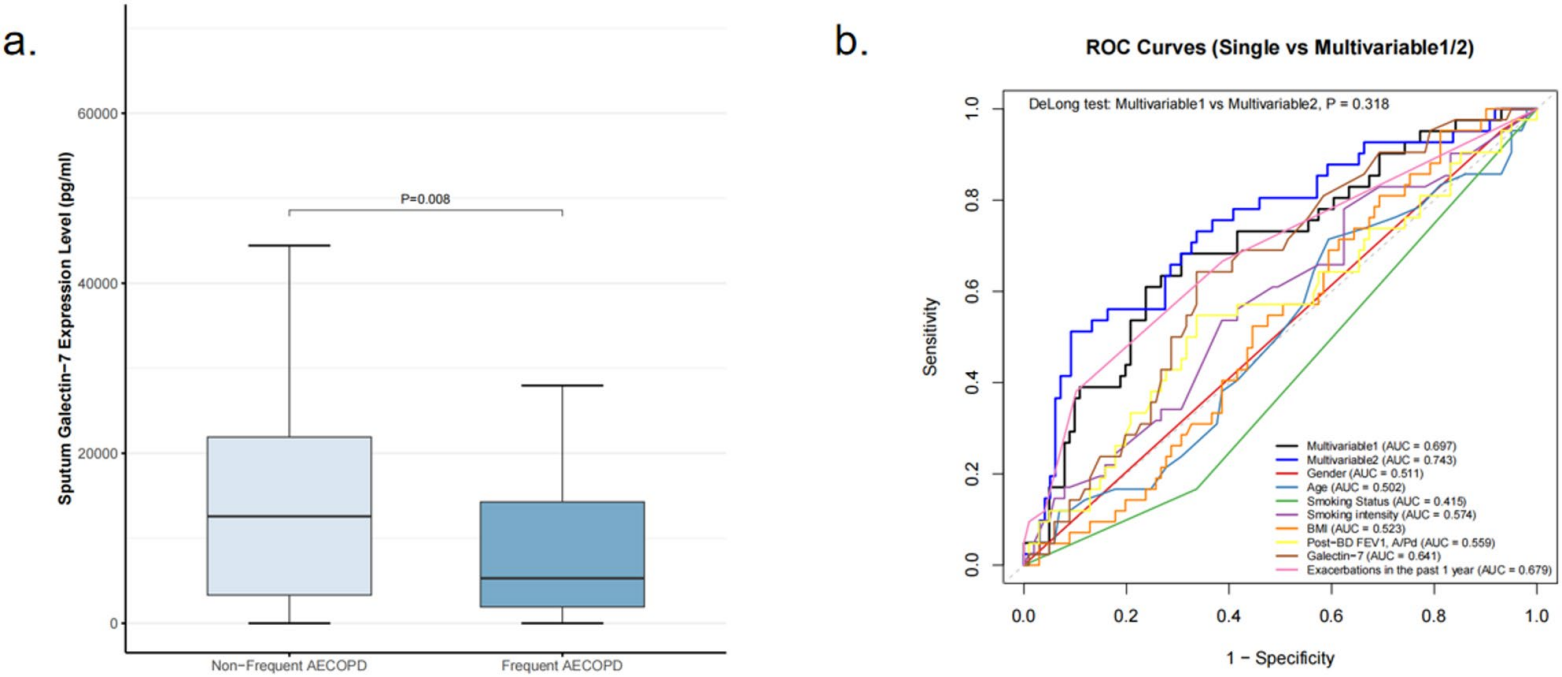
Sputum galectin-7 and future frequent acute exacerbations in COPD: (**a**) comparison of baseline sputum galectin-7 levels between frequent AECOPD and non-frequent AECOPD groups; (**b**) prediction of future frequent acute exacerbations using age, sex, smoking status, BMI, lung function, and other confounders, exacerbation history, and sputum galectin-7, with performance comparison between Multivariable Model 1 (confounders + galectin-7) and Multivariable Model 2 (confounders + exacerbation history)

A total of 143 patients completed the one-year follow-up for acute exacerbations. Baseline sputum galectin-7 levels were significantly lower in COPD patients who experienced acute exacerbations during the follow-up period compared with those who did not (7348 [4851, 12573] vs. 14275 [9542, 17439], *P* = 0.025; Fig. 5, a). In multivariate Poisson regression analysis adjusting for potential confounding factors, sputum galectin-7 levels remained independently associated with the risk of acute exacerbations (RR = 0.843, 95% CI: 0.718–0.974, *P* = 0.028, Table 5). For every 10 ng/mL increase in sputum galectin-7, the relative risk of acute exacerbations decreased by approximately 15.7%. Furthermore, patients with frequent acute exacerbations during the one-year follow-up period had significantly lower sputum galectin-7 levels than those with infrequent exacerbations (5296 [2953, 10030] vs. 12573 [9542, 16332], *p* = 0.008; Fig. 6, a). In multivariate logistic regression analysis adjusting for confounding factors, sputum galectin-7 levels remained significantly associated with the risk of frequent future acute exacerbations (OR = 0.649, 95% CI: 0.424–0.923, *P* = 0.029, Table 6). For every 10 ng/mL increase in sputum galectin-7 levels, the risk of frequent future acute exacerbations decreased by approximately 35.1% (Table 6).

**Table 5:** Poisson regression analysis of factors associated with the number of acute exacerbations in COPD patients

Variables	Univariate analysis			Multivariate analysis		
	RR	95% CI	*P*	RR	95% CI	*P*
Gender	1.324	0.669–3.126	0.469			
Age, y	0.992	0.973–1.011	0.407			
Smoking status	0.733	0.49–1.067	0.117			
Smoking intensity, pack/years	1.008	1.002–1.013	**0.006**	1.007	1.001–1.012	**0.022**
BMI, kg/m2	0.971	0.925–1.018	0.229			
Post-BD FEV_1_% pred	0.991	0.983–0.998	**0.019**	0.997	0.988–1.005	0.441
Exacerbations in the past 1 year	1.155	1.049–1.253	**0.001**	1.124	1.012–1.227	**0.017**
galectin-7,10ng/ml	0.799	0.685–0.918	**0.003**	0.843	0.718–0.974	**0.028**

*P*-values < 0.05 are shown in boldface

**Table 6:** Logistic regression analysis of factors associated with the risk of frequent acute exacerbation in COPD patients

Variables	Univariate analysis			Multivariate analysis		
	OR	95% CI	*P*	OR	95% CI	*P*
Gender	1.489	0.342–10.29	0.629			
Age, y	1	0.957–1.044	0.986			
Smoking status	0.394	0.148–0.936	**0.045**	0.448	0.158–1.139	0.106
Smoking intensity, pack/years	1.01	0.997–1.024	0.145			
BMI, kg/m2	0.97	0.871–1.078	0.574			
Post-BD FEV_1_% pred	0.992	0.975–1.008	0.317			
Exacerbations in the past 1 year	1.926	1.346–2.895	**< ** **0.001**	1.898	1.309–2.87	**0.001**
galectin-7,10ng/ml	0.61	0.409–0.851	**0.008**	0.649	0.424–0.923	**0.029**

*P*-values < 0.05 are shown in boldface

To compare the predictive performance of sputum galectin-7 and a history of prior acute exacerbations for identifying patients at risk of future acute exacerbations, we constructed ROC models. In single-predictor ROC analyses, the AUCs were 0.611 for sputum galectin-7 and 0.630 for a history of prior exacerbations. Two multivariate models were then developed: Model 1(Multivariable 1 ), incorporating clinical confounders and sputum galectin-7, and Model 2(Multivariable 2), incorporating clinical confounders and a history of prior exacerbations. Model 1 achieved an AUC of 0.682, whereas Model 2 achieved an AUC of 0.730. DeLong’s test indicated no statistically significant difference between the two ROC curves (*P* = 0.159; Fig. 5, b).We performed a similar comparison for predicting frequent future acute exacerbations. In single-predictor analyses, the AUCs were 0.641 for sputum galectin-7 and 0.679 for prior exacerbations. In the multivariate setting, Model 1 (clinical confounders plus sputum galectin-7) achieved an AUC of 0.697, while Model 2 (clinical confounders plus prior exacerbations) achieved an AUC of 0.743. DeLong’s test again showed no significant difference between the two models (*P* = 0.318; Fig. 6, b).

## Discussion

To our knowledge, this is the first prospective study conducted in a Chinese population to characterize sputum galectin-7 levels in healthy subjects and patients across different GOLD lung function stages, and to evaluate their associations with clinical characteristics and prognosis. After adjusting for relevant covariates, the difference in sputum galectin-7 levels between healthy controls and GOLD I–II patients was no longer statistically significant. In contrast, sputum galectin-7 levels were significantly lower in GOLD III–IV patients compared to both healthy controls and GOLD I–II patients, suggesting that the decrease in galectin-7 levels mainly occurs in COPD patients with more severe airflow limitation. ROC curve analysis further demonstrated that sputum galectin-7 showed a moderate overall ability to discriminate COPD, with better discriminatory performance to GOLD III–IV patients than in those with GOLD I–II. These findings suggest that galectin-7 may be more suitable as a marker associated with disease severity rather than as an independent diagnostic biomarker.

Among COPD patients, sputum galectin-7 showed significant associations with lung function, symptom burden (CAT, mMRC), systemic inflammatory indices ( neutrophils, globulin, HsCRP), and several sputum cytokines. In addition, in the adjusted multivariable models, sputum galectin-7 levels and a history of prior exacerbations were independently associated with the risk of future acute exacerbations and frequent exacerbations. However, ROC curve comparisons suggested that the incremental predictive value of galectin-7 relative to prior exacerbation history was limited. There was no significant difference in predictive performance between the two markers for either future exacerbations or frequent exacerbations. Galectin-7 may be useful for patients subject to recall bias.

Multiple previous studies have confirmed that galectin-7 can exert protective effects in chronic inflammatory diseases. In psoriasis, galectin-7 alleviates inflammatory responses by suppressing the IL-17 A/MAPK pathway [27]. In systemic sclerosis, its downregulation is closely associated with disease progression and esophageal dysfunction [28]. In this study, we also observed a negative linear correlation between IL-17 A and galectin-7. Furthermore, lower levels of galectin-7 in sputum were associated with more severe airflow limitation, higher levels of airway IL and MMPs factors, greater symptom severity, more extensive emphysema, and an increased risk of future acute exacerbations. These findings, to some extent, support the possibility that galectin-7 may be involved in protective processes during disease progression and suggest a clinical association with the severity and prognosis of COPD. However, given the observational design of this study, it is not possible to determine the causal relationship or underlying mechanisms of galectin-7 in the pathogenesis of COPD.

Galectin-7 plays an important role in immune defense and the regulation of inflammatory balance. The study by Luo et al. confirmed that galectin-7 promotes Th1 polarization of CD4 + T cells and inhibits the TGF-β/Smad3 signaling pathway, thereby enhancing immune responses [29]. Meanwhile, Lin and colleagues demonstrated using HaCaT cell models that intracellular galectin-7 participates in autophagy and restricts bacterial proliferation, highlighting its role in antibacterial immunity [30]. Downregulated galectin-7 levels may weaken host immune defense, promoting bacterial colonization and infection. IL-17B is recognized for their roles in mucosal immune defense, particularly against extracellular bacterial and fungal infections [31–34]. We also observed a positive linear correlation between galectin-7 and IL-17B. Whether galectin-7 influences the risk of acute exacerbations through the aforementioned pathways remains unclear. The results of this study provide preliminary clues for this hypothesis, but further research is needed to verify it. Furthermore, we also found that decreased sputum galectin-7 levels were accompanied by elevated serum globulin levels. Increased serum globulin typically reflects persistent immune activation or chronic inflammatory responses and has been associated with disease severity, lung function impairment, and increased mortality in previous studies [34]. In our cohort, this pattern may indicate that reduced sputum galectin-7 levels are associated with a broader systemic inflammatory or immune activation phenotype. However, given the observational nature of the study, neither directionality nor causality can be established, and residual confounding factors (e.g., infection burden, comorbidities, or medication effects) may partially account for these associations. Furthermore, as multiple biomarkers and clinical variables may co-vary with disease severity, these findings should be interpreted with caution and regarded as exploratory.

Sputum collection represents a non-invasive detection method that facilitates clinical application. This study utilized non-invasive sputum samples, demonstrating strong translational potential. Our findings confirm the association between sputum galectin-7 levels and clinical characteristics in COPD patients, as well as its independent predictive value for future acute exacerbation risk. We recommend that clinicians consider early intervention and intensified treatment when sputum galectin-7 levels decrease, in order to reduce future exacerbation risk and alleviate healthcare burden. Dynamic monitoring of sputum galectin-7 levels in COPD patients may provide valuable insights for tracking disease activity and serve as an early warning indicator for acute exacerbations.

This study has several limitations. First, as a single-center investigation with relatively concentrated sample sources, selection bias may exist. Importantly, the study population was predominantly male (> 90%). Although sex was included as a covariate in our statistical models, this adjustment cannot fully address the limited external validity and generalizability resulting from the underrepresentation of female patients. Therefore, the applicability of our findings to female COPD patients remains uncertain and warrants further validation in multicenter cohorts with a more balanced sex distribution or a larger sample of female participants. Second, our analysis primarily examined the associations between galectin-7 levels and clinical indicators, without conducting mechanistic validation at cellular or animal levels. This limits the ability to clarify specific pathways of galectin-7 within COPD inflammatory networks or immune regulation. Third, this study did not include sputum cytology classification data (including the proportion of epithelial cells); therefore, it was not possible to assess the relationship between sputum galectin-7 and inflammatory cell composition or airway epithelial remodeling. Future studies should incorporate standardized sputum cytological analyses to further validate this correlation and investigate whether galectin-7 could serve as a surrogate marker for airway epithelial remodeling. Fourth, given the exploratory nature of the biomarker analysis involving multiple cytokines, we did not apply corrections for multiple testing; therefore, the reported correlations should be considered hypothesis-generating and require replication in independent cohorts.

In summary, sputum galectin-7 represents a promising local biomarker, and its decreased levels are significantly associated with elevated inflammatory factors, disease severity, and future acute exacerbation risk in COPD patients. These findings highlight its potential utility as a novel biomarker for assessing disease stratification and prognosis in COPD.

## Supplementary Information

Supplementary Material 1.

Supplementary Material 2.

## Data Availability

The datasets analyzed in this study can be obtained from the corresponding authors upon reasonable request.
