# Supplementary material for: The relevance of sputum galectin-7 levels to clinical and prognostic factors in patients with chronic obstructive pulmonary disease: a prospective cohort study from China
Source: BMC Pulm Med. 2026 Feb 27;26:156. doi: 10.1186/s12890-026-04171-9 (PMC13049942; doi:10.1186/s12890-026-04171-9)
Supplement: Supplementary file 1 — Supplementary Material 1. [file 12890_2026_4171_MOESM1_ESM.docx]

| **(pg/ml)** | **Cntrl** | **Std7** | **Std6** | **Std5** | **Std4** | **Std3** | **Std2** | **Std1** |
| --- | --- | --- | --- | --- | --- | --- | --- | --- |
| MMP-1 | 0 | 55 | 165 | 494 | 1,481 | 4,444 | 13,333 | 40,000 |
| MMP-8 | 0 | 14 | 41 | 123 | 370 | 1,111 | 3,333 | 10,000 |
| MMP-9 | 0 | 27 | 82 | 247 | 741 | 2,222 | 6,667 | 20,000 |
| MMP-13 | 0 | 14 | 41 | 123 | 370 | 1,111 | 3,333 | 10,000 |
| IL-1a | 0 | 3 | 8 | 25 | 74 | 222 | 667 | 2,000 |
| IL-1b | 0 | 1 | 4 | 12 | 37 | 111 | 333 | 1,000 |
| IL-1ra | 0 | 3 | 8 | 25 | 74 | 222 | 667 | 2,000 |
| IL-5 | 0 | 5 | 16 | 49 | 148 | 444 | 1,333 | 4,000 |
| IL-6 | 0 | 3 | 8 | 25 | 74 | 222 | 667 | 2,000 |
| IL-6R | 0 | 14 | 41 | 123 | 370 | 1,111 | 3,333 | 10,000 |
| IL-7 | 0 | 5 | 16 | 49 | 148 | 444 | 1,333 | 4,000 |
| IL-8 | 0 | 1 | 2 | 6 | 19 | 56 | 167 | 500 |
| IL-10 | 0 | 5 | 16 | 49 | 148 | 444 | 1,333 | 4,000 |
| IL-13 | 0 | 1 | 4 | 12 | 37 | 111 | 333 | 1,000 |
| IL-16 | 0 | 7 | 21 | 62 | 185 | 556 | 1,667 | 5,000 |
| IL-17 | 0 | 5 | 16 | 49 | 148 | 444 | 1,333 | 4,000 |
| IL-17F | 0 | 137 | 412 | 1,235 | 3,704 | 11,111 | 33,333 | 100,000 |
| IL-17B | 0 | 55 | 165 | 494 | 1,481 | 4,444 | 13,333 | 40,000 |
| IL-2 Ra | 0 | 14 | 41 | 123 | 370 | 1,111 | 3,333 | 10,000 |
| IL-2 Rb | 0 | 137 | 412 | 1,235 | 3,704 | 11,111 | 33,333 | 100,000 |
| IL-18 BPa | 0 | 82 | 247 | 741 | 2222 | 6667 | 20000 | 60000 |
| IL-28A | 0 | 14 | 41 | 123 | 370 | 1111 | 3333 | 10000 |
| Galectin-7 | 0 | 137 | 412 | 1,235 | 3,704 | 11,111 | 33,333 | 100,000 |

Supplemental Table 1. Concentrations of MMP Family, Interleukin Family and Galectin-7 Protein in Standard Products of Various Gradients (pg/ml)
