# Supplementary material for: The relevance of sputum galectin-7 levels to clinical and prognostic factors in patients with chronic obstructive pulmonary disease: a prospective cohort study from China
Source: BMC Pulm Med. 2026 Feb 27;26:156. doi: 10.1186/s12890-026-04171-9 (PMC13049942; doi:10.1186/s12890-026-04171-9)

**Research Methods**

Univariate receiver operating characteristic (ROC) curve analyses were conducted for 23 sputum supernatant inflammatory factors—including the IL family, MMP family, and Galectin-7—comparing healthy subjects versus COPD patients and MCOPD versus SCOPD patients. The four inflammatory factors with the highest AUC values from the univariate ROC analyses in each comparison were selected to construct a multivariable ROC model.

**Tables**

**Supplementary Table 1. Performance indicators (AUC, threshold, sensitivity, specificity) of ROC curves for 23 biomarkers differentiating healthy individuals from those with GOLD I-II.**

| Variable | AUC | threshold | sensitivity | specificity |
| --- | --- | --- | --- | --- |
| IL-17F | 0.72 | 52.122 | 0.58 | 0.78 |
| MMP-13 | 0.673 | 261.924 | 0.667 | 0.64 |
| IL-6 | 0.669 | 83.01 | 0.79 | 0.54 |
| Galectin-7 | 0.665 | 20952.578 | 0.716 | 0.6 |
| IL-18 BPa | 0.661 | 196.153 | 0.358 | 0.94 |
| IL-28A | 0.652 | 5.368 | 0.519 | 0.76 |
| MMP-1 | 0.637 | 2044.174 | 0.284 | 0.96 |
| IL-10 | 0.635 | 9.097 | 0.383 | 0.86 |
| IL-1a | 0.627 | 83.01 | 0.815 | 0.44 |
| IL-16 | 0.611 | 490.585 | 0.407 | 0.84 |
| IL-5 | 0.607 | 9.097 | 0.284 | 0.9 |
| IL-2 Ra | 0.597 | 17.14 | 0.704 | 0.58 |
| IL-17 | 0.572 | 5.368 | 0.63 | 0.5 |
| IL-8 | 0.571 | 632.604 | 0.198 | 0.96 |
| IL-17B | 0.562 | 1054.569 | 0.506 | 0.7 |
| IL-2 Rb | 0.55 | 1875.989 | 0.617 | 0.52 |
| IL-13 | 0.549 | 7.275 | 0.272 | 0.9 |
| IL-1b | 0.544 | 21.923 | 0.506 | 0.66 |
| MMP-9 | 0.541 | 5476.378 | 0.185 | 0.94 |
| IL-1ra | 0.539 | 235.591 | 0.691 | 0.42 |
| IL-7 | 0.529 | 12.796 | 0.296 | 0.84 |
| IL-6R | 0.525 | 542.589 | 0.383 | 0.74 |

**Supplementary Table 2. Performance metrics (AUC, threshold, sensitivity, specificity) of ROC curves for 23 biomarkers differentiating healthy individuals from those with GOLD III-IV**

| Variable | AUC | threshold | sensitivity | specificity |
| --- | --- | --- | --- | --- |
| Galectin-7 | 0.834 | 13016.769 | 0.739 | 0.78 |
| IL-6 | 0.8 | 248.634 | 0.725 | 0.8 |
| IL-10 | 0.788 | 6.23 | 0.681 | 0.76 |
| IL-5 | 0.782 | 4.97 | 0.681 | 0.8 |
| IL-17B | 0.78 | 1010.766 | 0.812 | 0.72 |
| MMP-1 | 0.75 | 2044.174 | 0.507 | 0.96 |
| IL-16 | 0.746 | 515.621 | 0.58 | 0.86 |
| IL-17 | 0.745 | 7.275 | 0.87 | 0.6 |
| MMP-9 | 0.7 | 13016.769 | 0.623 | 0.72 |
| IL-1b | 0.674 | 65.435 | 0.565 | 0.74 |
| MMP-8 | 0.669 | 10260.59 | 0.551 | 0.72 |
| IL-17F | 0.656 | 5.579 | 0.58 | 0.68 |
| IL-28A | 0.651 | 0.815 | 0.826 | 0.44 |
| IL-6R | 0.612 | 515.621 | 0.507 | 0.7 |
| MMP-13 | 0.609 | 261.924 | 0.551 | 0.64 |
| IL-8 | 0.593 | 632.604 | 0.261 | 0.96 |
| IL-18 BPa | 0.59 | 5.579 | 0.565 | 0.62 |
| IL-7 | 0.588 | 7.275 | 0.522 | 0.66 |
| IL-13 | 0.587 | 5.579 | 0.319 | 0.86 |
| IL-2 Ra | 0.573 | 11.694 | 0.681 | 0.6 |
| IL-1ra | 0.56 | 153.638 | 0.507 | 0.64 |
| IL-2 Rb | 0.527 | 1758.231 | 0.681 | 0.48 |

**Supplementary Table 3. Performance metrics (AUC, threshold, sensitivity, specificity) of ROC curves for 23 biomarkers differentiating healthy individuals from those with COPD.**

| Variable | AUC | threshold | sensitivity | specificity |
| --- | --- | --- | --- | --- |
| Galectin-7 | 0.743 | 20952.578 | 0.793 | 0.6 |
| IL-6 | 0.73 | 83.01 | 0.84 | 0.54 |
| IL-10 | 0.705 | 6.23 | 0.573 | 0.76 |
| IL-17F | 0.69 | 5.368 | 0.627 | 0.68 |
| MMP-1 | 0.689 | 2044.174 | 0.387 | 0.96 |
| IL-5 | 0.687 | 4.97 | 0.513 | 0.8 |
| IL-16 | 0.673 | 490.585 | 0.487 | 0.84 |
| IL-17B | 0.662 | 1054.569 | 0.653 | 0.7 |
| IL-17 | 0.652 | 7.275 | 0.673 | 0.6 |
| IL-28A | 0.651 | 5.368 | 0.487 | 0.76 |
| MMP-13 | 0.643 | 261.924 | 0.613 | 0.64 |
| IL-18 BPa | 0.628 | 5.368 | 0.587 | 0.62 |
| IL-2 Ra | 0.586 | 11.694 | 0.68 | 0.6 |
| IL-8 | 0.582 | 632.604 | 0.227 | 0.96 |
| IL-1a | 0.573 | 37.17 | 0.513 | 0.64 |
| MMP-9 | 0.57 | 16885.544 | 0.187 | 1 |
| MMP-8 | 0.568 | 10260.59 | 0.42 | 0.72 |
| IL-13 | 0.566 | 6.23 | 0.287 | 0.88 |
| IL-6R | 0.565 | 515.621 | 0.46 | 0.7 |
| IL-1b | 0.556 | 126.625 | 0.24 | 0.92 |
| IL-7 | 0.556 | 12.796 | 0.307 | 0.84 |
| IL-1ra | 0.549 | 153.638 | 0.473 | 0.64 |

**Supplementary Table 4.Performance indicators (AUC, threshold, sensitivity, specificity) of ROC curves for 23 biomarkers used to differentiate GOLD I-II from GOLD III-IV**

| Variable | AUC | threshold | sensitivity | specificity |
| --- | --- | --- | --- | --- |
| Galectin-7 | 0.722 | 8438.948 | 0.667 | 0.741 |
| MMP-9 | 0.704 | 12208.815 | 0.681 | 0.679 |
| IL-6 | 0.7 | 222.215 | 0.739 | 0.63 |
| IL-17B | 0.693 | 1010.766 | 0.812 | 0.531 |
| IL-1b | 0.686 | 71.098 | 0.536 | 0.802 |
| MMP-8 | 0.68 | 7697.056 | 0.942 | 0.309 |
| IL-17 | 0.677 | 7.275 | 0.87 | 0.494 |
| IL-5 | 0.665 | 6.23 | 0.652 | 0.667 |
| IL-10 | 0.655 | 4.97 | 0.754 | 0.494 |
| IL-16 | 0.655 | 363.781 | 0.754 | 0.531 |
| MMP-1 | 0.634 | 1580.301 | 0.594 | 0.691 |
| IL-1a | 0.611 | 56.335 | 0.565 | 0.704 |
| IL-6R | 0.589 | 802.069 | 0.261 | 0.901 |
| IL-18 BPa | 0.582 | 180.218 | 0.87 | 0.358 |
| IL-17F | 0.561 | 154.069 | 0.638 | 0.506 |
| MMP-13 | 0.557 | 276.171 | 0.493 | 0.654 |
| IL-7 | 0.551 | 1.189 | 0.928 | 0.173 |
| IL-13 | 0.528 | 0.217 | 0.971 | 0.123 |
| IL-1ra | 0.526 | 135.28 | 0.449 | 0.691 |
| IL-8 | 0.526 | 153.638 | 0.986 | 0.123 |
| IL-28A | 0.52 | 7.093 | 0.638 | 0.481 |
| IL-2 Ra | 0.505 | 163.466 | 0.232 | 0.889 |

Supplementary figure 1. **ROC Curve Analysis of Diagnostic Performance for Distinguishing Different COPD Stages and Healthy Controls: Comparison of Sputum Galectin-7, Cytokines. a** ROC curve for distinguishing GOLD I-II from healthy controls. **b** ROC curve for distinguishing GOLD III-IV from healthy controls. **c** ROC curve for distinguishing overall COPD from healthy controls. **d** ROC curve for distinguishing GOLD I-II from GOLD III-IV.


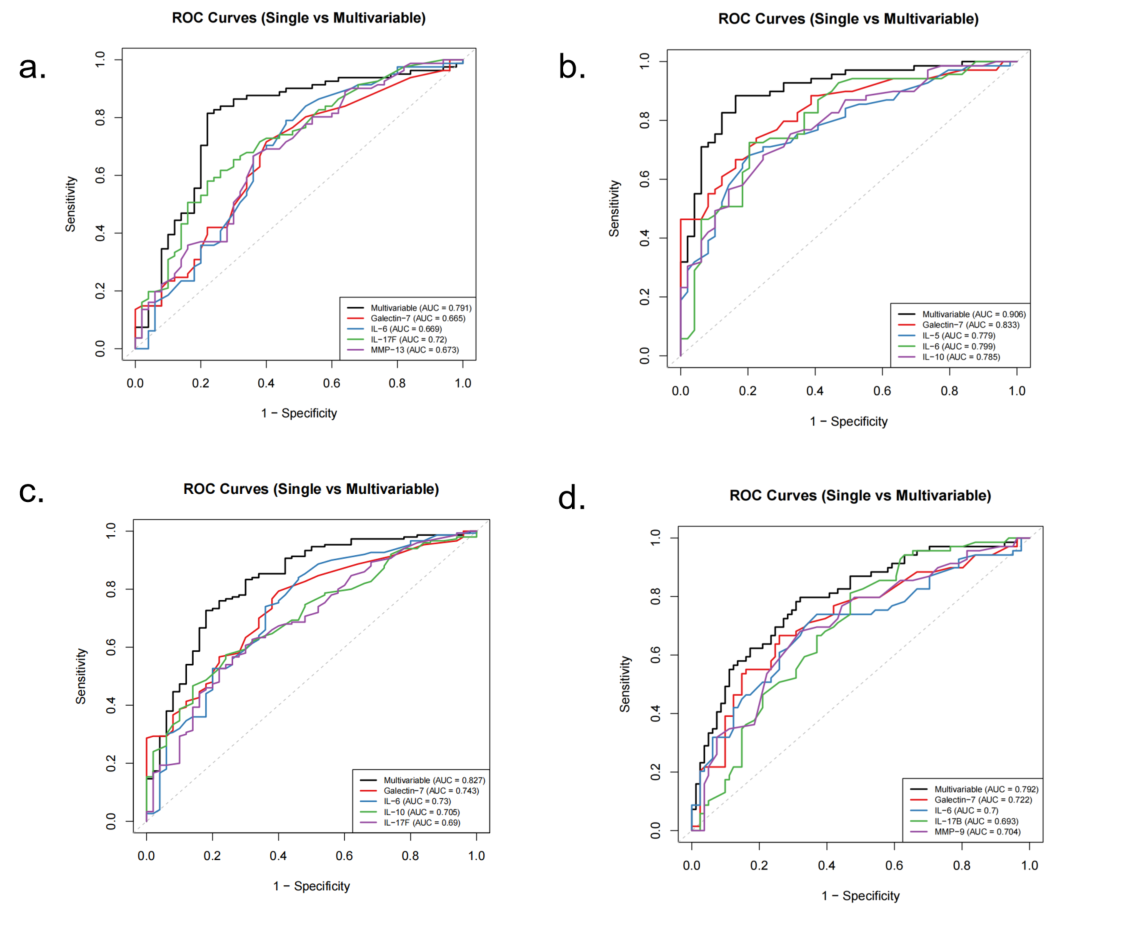

Supplement: Supplementary file 2 — Supplementary Material 2. [file 12890_2026_4171_MOESM2_ESM.docx]
